# High Salt Intake Enhances the REVERSIR-Induced Recovery of Blood Pressure After Angiotensinogen siRNA Treatment

**DOI:** 10.1161/HYPERTENSIONAHA.125.26638

**Published:** 2026-05-06

**Authors:** Edwyn O. Cruz-López, Richard van Veghel, Ingrid M. Garrelds, Anne Kasper, Kelly Wassarman, Ho-Chou Tu, Ivan Zlatev, A.H. Jan Danser

**Affiliations:** Division of Pharmacology and Vascular Medicine, Department of Internal Medicine, Erasmus MC, University Medical Center, Rotterdam, the Netherlands (E.O.C.-L., R.V., I.M.G., A.H.J.D.).; Alnylam Pharmaceuticals, Cambridge, MA (A.K., K.W., H.-C.T., I.Z.).

**Keywords:** animals, blood pressure, hypotension, sepsis, technology

GalNAc (N-acetylgalactosamine)-conjugated small interfering RNA (siRNA) targeting liver *Agt* mRNA has emerged as a promising strategy for long-lasting (>6 months after 1 injection) renin-angiotensin system (RAS) suppression.^1,2^ Yet, there might be conditions requiring acute RAS reactivation, such as sepsis or hemorrhage. In spontaneously hypertensive rats (SHRs) treated with angiotensinogen (AGT) siRNA, the recently developed REVERSIR (Reversal of siRNA Silencing, RVR) technology enabled full recovery of AGT synthesis and blood pressure within 5 to 7 days.^3^ To better reflect the low blood pressures observed in sepsis or hemorrhage, in the present study we treated normotensive Wistar Kyoto rats (WKYs) with AGT siRNA during salt depletion (low salt) and subsequently applied RVR-AGT, both with and without high salt (HS), given that HS also restored blood pressure in salt-depleted SHR treated with AGT siRNA.^4^ All experiments were performed under the regulation and approval of the Animal Care Committee of the Erasmus MC (protocol number SP2300200), using the experimental setup shown in Figure [A]. The data that support the findings of this study are available from the corresponding author upon reasonable request.

**Figure.**
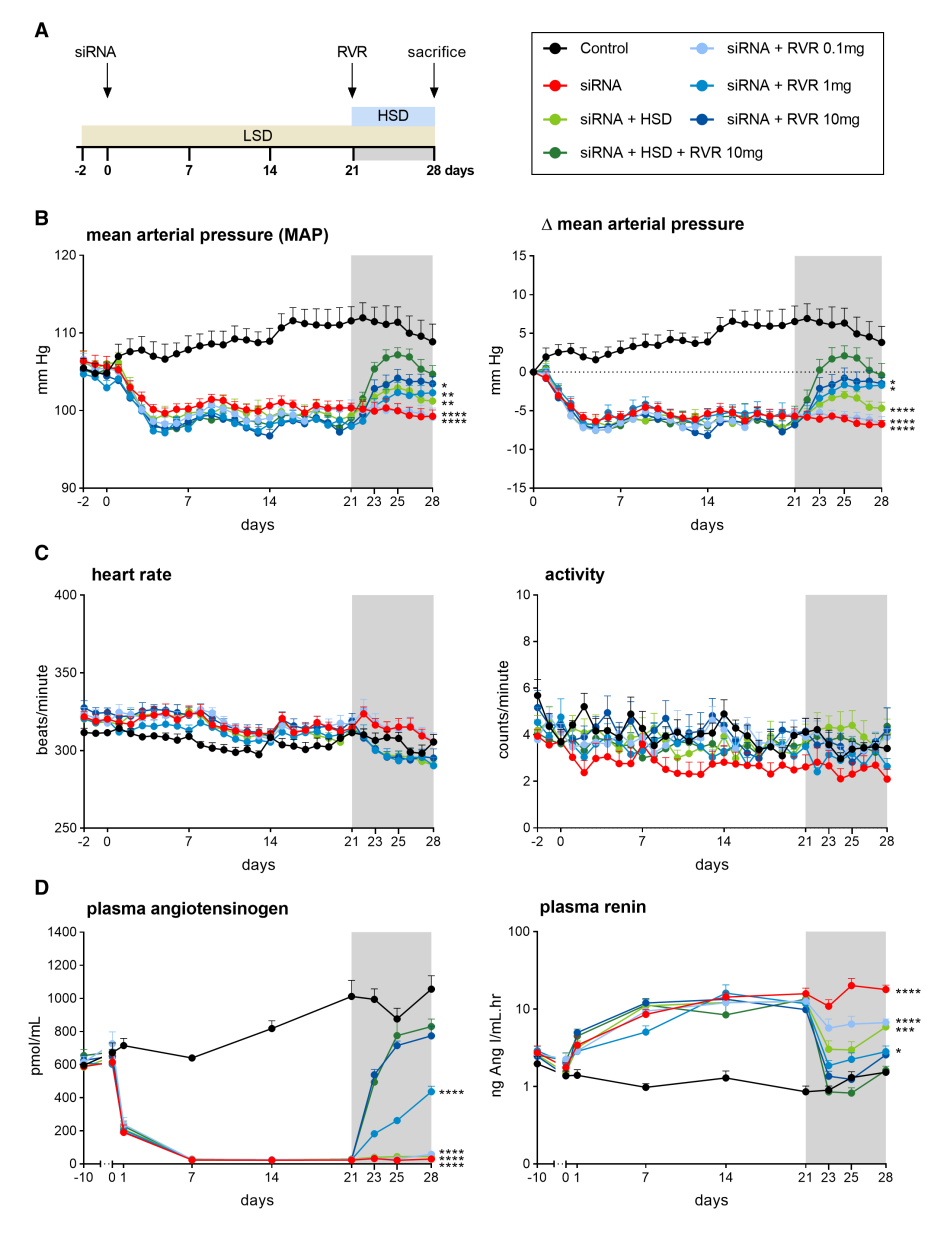
**RVR (Reversal of siRNA Silencing)-AGT (angiotensinogen) dose-dependently reverses the blood pressure reduction induced by 10 mg/kg AGT small interfering RNA (siRNA) in Wistar Kyoto (WKY) rats receiving a low-salt diet (LSD), and the reversal is enhanced when simultaneously switching to a high-salt diet (HSD). A**, Experimental setup. Ten-week-old male Wistar Kyoto rats (weight 310–330 g; Charles River Laboratories, Sulzfeld, Germany) were maintained on a 12-hour light/dark cycle with access to a diet containing 0.2% sodium (Ssniff-Spezialdiäten-GmbH, Soest, Germany) and water ad libitum. After 2 weeks, the diet was switched to a LSD (<0.03% Na^+^ purified diet; Ssniff-Spezialdiäten GmbH). One week later, rats received a subcutaneous injection of either vehicle (control, n=7) or AGT siRNA (10 mg/kg; n=44). At 16 weeks of age, the rats that had been injected with AGT siRNA received vehicle (n=7), RVR-AGT via subcutaneous injection at doses of 0.1 (n=7), 1 (n=8), or 10 mg/kg (n=8), HSD (4% Na^+^ purified diet, Ssniff-Spezialdiäten GmbH; n=7), or HSD + RVR (10 mg/kg; n=7). They were then monitored for 1 more week, after which they were sacrificed. Blood pressure, heart rate, and activity were measured by radiotelemetry transmitters (HD-S10, Data Sciences International, St. Paul, MN), implanted 2 weeks before the start of treatment. Inhaled anesthesia with isoflurane was applied during radiotelemetry transmitter and minipump implantation. Blood samples for the measurement of renin and AGT by enzyme-kinetic assay were collected by venipuncture from the lateral tail vein. At the end of the treatment period, rats were anaesthetized by inhalation of isoflurane and exsanguinated. Blood was collected in EDTA tubes (MiniCollect 1 mL K3EDTA, Greiner Bio-One GmBh, Kremsmünster, Austria), and plasma was obtained after centrifugation for 10 min, and stored at −80 °C. **B**, Mean arterial pressure (MAP) and delta MAP, (**C**) heart rate and activity, (**D**) plasma angiotensinogen and plasma renin. Data are mean±SEM. Nonnormally distributed data were log-transformed before analysis. Data were analyzed using 1-way ANOVA and mixed linear models, with treatment and time as fixed effects. When ANOVA indicated significance (*P*<0.05), post hoc tests were conducted (Dunnett test for comparisons vs control). All analyses were performed using Prism, version 9.0.0 (GraphPad Software, Inc, La Jolla, CA). *P<0.05, **P<0.01, ***P<0.001,****P<0.0001 vs control.

Baseline mean arterial blood pressure (MAP) in normotensive Wistar Kyoto rats was 105±3 mm Hg. The combination of low salt and AGT siRNA administration lowered MAP by 12 mm Hg and suppressed AGT by >95% (Figure [B] and [D]). Renin levels increased >11-fold (Figure [D]). Maximum changes in blood pressure were achieved after 1 week of siRNA treatment and remained stable until the animals were sacrificed at week 4. AGT siRNA did not affect heart rate or activity (Figure [C]). RVR-AGT, when given at 3 weeks after AGT siRNA injection at a dose of 0.1 mg/kg, was without effect. Yet, when given at doses of 1 and 10 mg/kg, RVR-AGT fully restored MAP to baseline levels within 2 to 3 days. Only the 10 mg/kg dose allowed AGT and renin to return to baseline levels, while the 1 mg/kg dose reversed the effect of AGT siRNA on AGT and renin by ≈50%. HS alone did not affect AGT, but partially normalized renin and MAP. When HS was combined with RVR-AGT 10 mg/kg, both AGT and renin normalized fully, and the accompanying MAP rise equaled the sum of the individual effects of these treatments. Only in the rats exposed to HS + RVR-AGT 10 mg/kg was MAP not different from that in the control group.

This study is the first to demonstrate that RVR-AGT reverses the effects of AGT siRNA in a hypotensive model. A switch to HS only partially restored blood pressure. This is different from salt-depleted SHR treated with AGT siRNA, where HS fully allowed blood pressure to return to baseline.^4^ Remarkably, when HS was given on top of 10 mg/kg RVR-AGT in salt-depleted WKY rats, the effect of the 2 treatments equaled the sum of both separate effects. In fact, under those conditions, MAP exceeded baseline levels and no longer differed from that in vehicle-treated WKY rats on a low-salt diet. Salt-depleted WKY rats receiving vehicle displayed a modest MAP rise over the 4 weeks of the study, most likely due to the upregulation of the RAS and sympathetic nervous system occurring under such conditions.^5^ Importantly, because of the additive effects, HS + RVR-AGT allowed MAP to reach baseline levels earlier than each separate treatment. If anything, HS more rapidly lowered renin, and this may have helped to upregulate AGT faster.^4^ Additional effects of HS explaining its blood pressure effects concern (transient) fluid retention and sodium-dependent modulation of vascular tone.

In line with earlier studies in SHR,^3^ RVR-AGT normalized MAP within 5 to 7 days at doses ≥1 mg/kg. Notably, MAP even returned to baseline when circulating AGT had reached only 50% of its pre-AGT siRNA levels. This indicates that partial recovery of hepatic AGT, together with renin levels remaining elevated, is sufficient to reestablish RAS activity. This reestablishment will only work when AGT levels are in a range that allows high renin levels to generate more angiotensin I. At very low AGT levels, renin elevation might even exert the opposite effect, and induce near-depletion of AGT, resulting in RAS annihilation. Indeed, this was observed in SHR treated with AGT siRNA + valsartan. This combination resulted in such high renin levels that AGT virtually disappeared, allowing angiotensin levels to become undetectable.^1^ Consequently, a synergistic effect on MAP was observed.^1^ This raises the possibility that the return of blood pressure in patients in whom AGT was not as aggressively lowered with the initial AGT siRNA treatment, as in the present WKY model, might be more rapid after RVR-AGT application.

In conclusion, a rapid return of blood pressure in hypotensive animals exposed to AGT siRNA can be obtained with RVR-AGT, and this effect is further amplified in combination with HS. In clinical settings where AGT siRNA is used for chronic blood pressure control, we should now evaluate whether RVR-AGT with or without HS could provide a selective and controlled method to reverse AGT depletion, enabling rapid RAS reactivation and blood pressure restoration after acute hypotensive events.

## Article Information

### Disclosures

A. Kasper, K. Wassarman, H.-C. Tu, and I. Zlatev are employees of Alnylam Pharmaceuticals. A.H.J. Danser received a grant from Alnylam Pharmaceuticals, which has partially supported this work. The other authors report no conflicts.
